# Effect of extensive artery isolation during robotic-assisted partial nephrectomy on blood pressure of patients with poorly controlled hypertension: a preliminary study

**DOI:** 10.1007/s11255-022-03384-1

**Published:** 2022-10-30

**Authors:** Xin Wang, Xiaozhi Zhao, ChangWei Ji, Guangxiang Liu, Xiaogong Li, Hongqian Guo

**Affiliations:** Department of Urology, Drum Tower Hospital, Institute of Urology, Medical School of Nanjing University, Nanjing University, 321 Zhongshan Rd., Nanjing, 210008 Jiangsu People’s Republic of China

**Keywords:** Kidney neoplasms, Hypertension, Renal denervation, Sympathetic nerve

## Abstract

**Purpose:**

To investigate whether extensive renal artery isolation during robotic-assisted partial nephrectomy (RAPN) for renal cell carcinoma (RCC) affects blood pressure (BP) of patients with poorly controlled hypertension.

**Methods:**

We included 60 patients diagnosed with poorly controlled hypertension who underwent RAPN by an experienced surgeon. The renal artery of the treated kidney was sufficiently isolated. Systolic BP (SBP), diastolic BP (DBP) and antihypertensive medication information were obtained at baseline and 3- and 6-month follow-up after surgery. Primary endpoints were changes in BP, and medications. Predictors of SBP reduction at 3 months were assessed by multivariable logistic regression.

**Results:**

All 60 RAPN procedures were successful, with no major intra- or postoperative complications. Mean SBP and DBP decreased significantly at 3 months after surgery (SBP, −7.8 ± 6.3 mmHg, *P* < 0.001; DBP, −4.2 ± 6.4 mmHg, *P* = 0.01). SBP and DBP did not differ between 3- and 6-month follow-up. The mean number of BP medications prescribed was lower at 3 months than baseline (1.7 ± 1.0 vs 2.1 ± 1.0, *P* = 0.016). The only significant predictor of SBP reduction at 3 months was baseline SBP.

**Conclusions:**

Renal denervation with extensive renal artery isolation during RAPN may improve BP control among patients with poorly controlled hypertension in short term.

## Introduction

Renal cell carcinoma (RCC) represents approximately 90% of all kidney malignancies. The incidence has shown an increasing trend worldwide [1]. The incidence rates tend to be elevated in older individuals, with a peak incidence at age 60–70 years. Smoking, obesity, and hypertension are important etiologic factors [2]. Partial nephrectomy (PN) has been the standard treatment for small, organ-confined RCC, and robotic-assisted PN (RAPN) has been widely applied.

Hypertension is an important global health issue because of its high prevalence and is a pivotal risk factor of cardiovascular disease and chronic kidney disease [3]. Moreover, numerous studies, including several large, prospective cohort studies, proposed a possible relation between hypertension and RCC [4, 5]. Despite the great progress in pharmaceutical treatment, many hypertensive patients still have poor blood pressure (BP) control. Renal denervation (RDN) has been a promising treatment for resistant hypertension in the past decade. The mechanism for RDN to treat resistant hypertension is breaking renal sympathetic nerves (RSNs) around renal arteries, thus decreasing autonomic sympathetic tone, renin release and renal tubular reabsorption [6].

During RAPN, the renal hilum is identified and exposed and the renal artery is isolated to prepare for temporary clamping. Then the kidney is separated to expose the tumor margin fully for tumor resection and reconstruction. On the basis of the effect of RDN, we hypothesized that thorough peri-arterial tissue stripping may affect RSNs and lead to altered BP. The aim of the present study was to investigate the effect of RAPN with relatively complete isolation of the renal artery on BP in patients with poorly controlled hypertension.

## Materials and methods

### Patient population

**Fig. 1:**
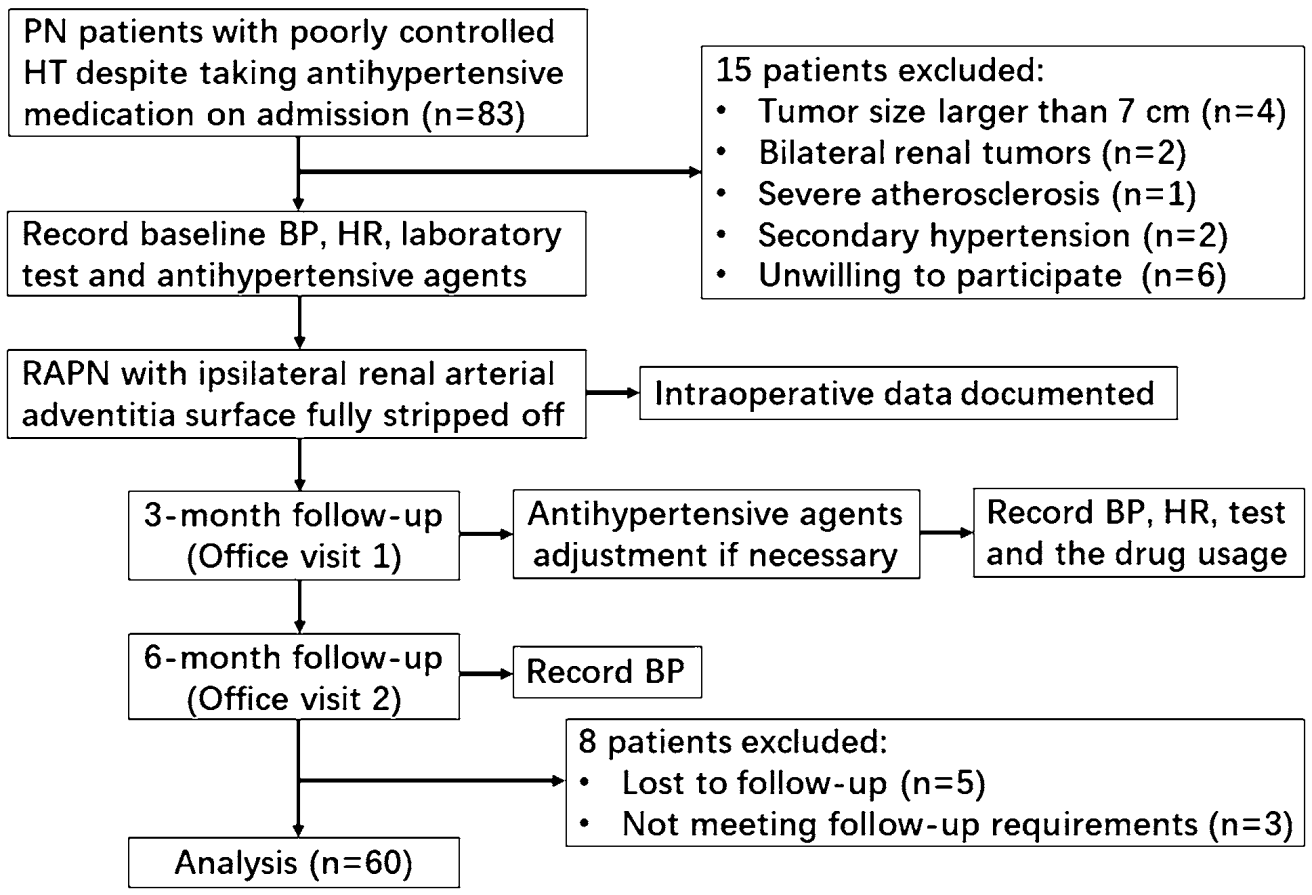
Study flow chart. *PN* partial nephrectomy, *HT* hypertension, *BP* blood pressure, *HR* heart rate, *RAPN* robotic-assisted partial nephrectomy

We prospectively investigated consecutive patients with kidney neoplasms and poorly controlled hypertension who underwent RAPN in our center between June 2018 and March 2020. The inclusion criteria were age between 25 and 85 years, indication for RAPN, and a history of hypertension and taking antihypertensive medications but with poor BP control [systolic BP (SBP) ≥ 140 mmHg and/or diastolic BP (DBP) ≥ 90 mmHg] at hospital admission. We excluded patients with bilateral renal tumors, tumors larger than 7 cm and severe atherosclerosis. Eligible patients underwent comprehensive assessments, which ruled out secondary hypertension (Fig. 1). The study was approved by the medical ethical committee of Nanjing Drum Tower hospital and were in accordance with the Helsinki Declaration. All patients gave their informed consent.

### Surgical technique

**Fig. 2:**
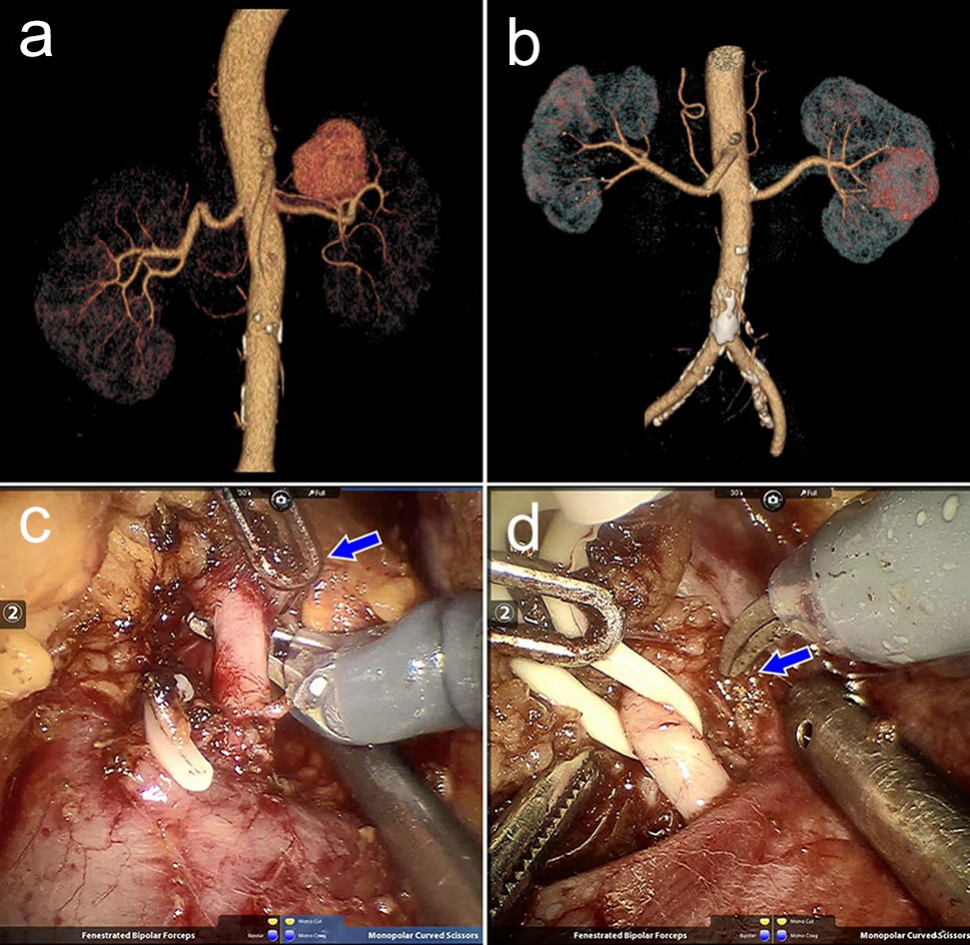
Representative renal arteries in CTAs and surgery videos. **a** A small accessory renal artery arising from the abdominal aorta feeds the left kidney with a mass at the superior pole. **b** There is only a main renal artery supplying the left kidney with a neoplasm in the middle zone. **c**, **d** The process of renal arteries dissection, during which the peri-arterial tissues were stripped, divided and cauterized to clear the arterial adventitia surface (blue arrow)

The surgical procedures were performed as described by a single experienced surgeon [7]. Briefly, the da Vinci Surgical System was used with a standard 3-arm approach, with fourth and fifth trocars for the assistant. The renal pedicle was identified and carefully isolated. Renal arteries were extensively isolated. The peri-arterial tissues were fully stripped, divided and cauterized, to clear the arterial adventitia surface (Fig. 2c, d). Accessory renal arteries and branches were also isolated under appropriate conditions (Fig. 2a, b). Then, the perinephric fat was separated to identify the tumor margin and exclude satellite lesions. Robotic scissors were used to excise the renal mass. The renal artery was clamped with vascular clips in most cases. For reconstruction, the parenchymal defect was closed using the single-layer renorrhaphy technique.

### BP assessments

SBP and DBP were assessed at baseline and at 3 and 6 months after RAPN, and changes were analyzed. Every BP value was calculated as the mean of three readings, with a 5-min rest between each measurement. BP measurements were collected twice a day, in the morning (9:00) and afternoon (16:00). Daily BP was the average of the two results. At least 2 days of BP measurements were obtained. The final BP value was calculated as the mean of the daily BPs.

### Medications

Detailed information on medication was recorded. If necessary, we invited cardiologists to adjust the background BP-lowering medications before surgery. The prescription after adjustment was considered the baseline medication, and baseline BP was calculated after the medication adjustment. Participants were instructed to maintain their medications at the adjusted doses from hospital discharge to the first visit at 3 months.

At 3 months, cardiologists in the cardiovascular specialist outpatient clinic of our center reconciled the medication, with blinding to the study. Changes in number of antihypertensive medications or dosage adjustment were obtained and documented. Furthermore, the re-modified prescription was maintained until the 6-month follow-up.

Drug enhancement was defined as upregulated number, dose or class of BP medication to improve BP control. Drug mitigation was defined as downregulated number, dose or class of medication intending to weaken the suppression effect.

### Other assessments

Preoperative urinary CT angiography was performed as routine and 3-D reconstructions of renal arteries were produced. Additional arteries (containing accessory renal artery and aberrant branches) were noted. Surgical videos were all archived and reviewed to record the number of arteries isolated.

Other data collected included patient demographics, personal history, nephrometry score, clinical data, surgical procedures, intraoperative or postoperative complications, perioperative outcomes and pathology. The estimated glomerular filtration rate (eGFR) was calculated using the Modification of Diet in Renal Diseases equation [8]. Plasma renin activity (PRA) and angiotensin II and aldosterone levels were measured at baseline and 3 months after surgery for patients without renin–angiotensin blocker drugs (anti-renin drugs, angiotensin-converting enzyme (ACE) inhibitor or angiotensin receptor blocking drugs) or antiadrenergic anti-hypertensive drugs. The specific time for blood sampling was 8:00 in the morning and the patient was asked to sit for half an hour before blood samples were taken.

### Statistical analysis

Data are presented as mean ± SD, median and interquartile range (IQR) for continuous variables or number (%) for categorical variables. Student *t* test was used to analyze continuous variables and chi-square test for categorical variables. Mean BP and other paired data at follow-up were compared with those at baseline by paired *t* test as appropriate. Univariable and multivariable stepwise logistic regression was used to explore potential factors relating to reduced BP. Variables with *P* < 0.15 on univariable analysis were included in multivariable models. Two-sided *P* < 0.05 was considered statistically significant. Statistical analysis was performed with SPSS v17.0 (SPSS Inc. Released 2008. SPSS Statistics for Windows, Version 17.0. Chicago: SPSS Inc.).

## Results

### Study population

Between January 2018 and November 2019, we included 60 patients (41 men): median age was 61 (IQR 52–66) years, median body mass index 25.5 (IQR 23.4–27.7) kg/m^2^ and median duration of hypertension 9.5 (IQR 5–14) years (Table 1).

**Table 1:** Baseline patient characteristics

Characteristics	Data
Patients, *n*	60
Age (years)	61 (14)
Sex	
Male	41 (68.3)
Female	19 (31.7)
BMI (kg/m^2^)	25.5 (4.3)
History of HTN (years)	9.5 (9)
Current smoking	20 (33.3)
CVD	11 (18.3)
Diabetes mellitus	17 (28.3)
Hyperlipidemia	10 (16.7)
SBP (mmHg)	146.3 ± 8.3
DBP (mmHg)	88.5 ± 8.6
Heart rate (bpm)	76.8 ± 9.9
Presence of additional artery	27 (33.3)
Solitary kidney	3 (5)

The results are presented as median and interquartile range (IQR), mean and standard deviation (SD), number and percent

*IQR* interquartile range, *BMI* body mass index, *HTN* hypertension, *CVD* cardiovascular disease, *SBP* systolic blood pressure, *DBP* diastolic blood pressure

Table 2 describes tumor and surgery characteristics. The median tumor size was 3.5 (IQR 2.5–4.5) cm. The median R.E.N.A.L score was 8 (IQR 6.5–10). All RAPN surgeries were completed without major intraoperative or perioperative complications. The median surgery time was 140 (IQR 121–160) min and median ischemia time 20 (IQR 15–25) min. The intraoperative estimated blood loss was 100 (IQR 50–200) ml, with no severe hemorrhage. The RCC subtype was clear cell in 45 (75%) cases.

**Table 2:** Tumor characteristics and perioperative parameters of robotic-assisted partial nephrectomy (RAPN)

Characteristics	Data
Tumors, *n*	60
Tumor side	
Left	32 (53.3)
Right	28 (46.7)
Solitary kidney	3 (5)
Tumor size (cm)	3.5 (2)
R.E.N.A.L. score	8 (3.5)
Surgery time (min)	140 (39)
WIT (min)	20 (10)
EBL (ml)	100 (150)
Perioperative complication	0 (0)
Not all arteries isolated	15 (25.0)
RCC subtypes	
Clear cell	45 (75)
Chromophobe	5 (8.3)
Papillary	9 (15)
Xp11.2 translocation	1 (1.7)

The results are presented as median and interquartile range (IQR), number and percent

*IQR* interquartile range, *WIT* warm ischemia time, *EBL* estimated blood loss, *RCC* renal cell carcinoma; *R.E.N.A.L.* R.E.N.A.L. Nephrometry Score

### Efficacy endpoints

**Fig. 3:**
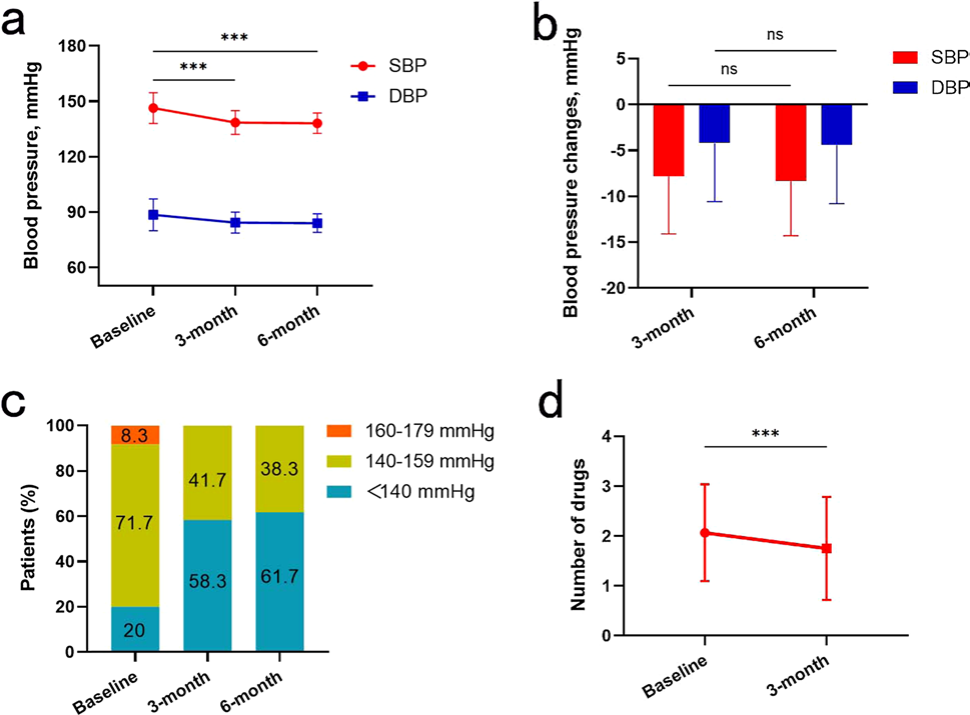
Changes in blood pressure (BP) and antihypertensive medication over time. **a** Office-measured systolic BP (SBP) and diastolic BP (DBP) at baseline, 3 months, and 6 months, respectively. **b** SBP and DBP reduction at 3 and 6 months after surgery. **c** Distribution of office-measured SBP at baseline, 3 months, and 6 months, showing a larger proportion of normal SBP postoperatively. **d** The number of antihypertensive drugs used before and 3 months after surgery

For all patients, mean baseline SBP/DBP was 146.3 ± 8.3/88.5 ± 8.6 mmHg, and 48 (80%) had high baseline SBP (≥ 140 mmHg) despite medication adjustment; the proportion of high SBP significantly decreased at the two follow-up visits (41.7% and 38.3%) (*P* < 0.05) (Fig. 3c). At 3 months, SBP and DBP had significantly decreased −7.8 ± 6.3 mmHg [95% confidence interval (CI) −9.5 to −6.2; *P* < 0.001] and −4.2 ± 6.4 mmHg (95% CI −5.9 to −2.6; *P* < 0.001), respectively (Fig. 3a). Although the mean BP was slightly lower at 6 than 3 months, we found no significant differences for SBP between 3 and 6 months (138.5 ± 6.4 vs 138.1 ± 5.5 mmHg, *P* = 0.079) or DBP (84.3 ± 5.7 vs 84.0 ± 5.0 mmHg, *P* = 0.066) (Fig. 3b). Heart rate (HR) did not significantly change between baseline and 3 months (76.8 ± 9.9 vs 75.2 ± 10.2 bpm, *P* = 0.298).

The mean baseline number of antihypertensive drugs was 2.1 ± 1.0 and the mean number of antihypertensive medications prescribed was significantly reduced at 3 months (−0.4 drugs; *P* = 0.0006) (Fig. 3d). The number of drugs was increased for 4 (6.7%) patients, but 24 (40%) showed drug mitigation, 5 with normal BP and no antihypertensive medication (Table 3).

**Table 3:** Biochemical characteristics and use of antihypertensive medication and before and 3 months after surgery

BP medications and renal function	Baseline (*n* = 60)	3-month (*n* = 60)	*P* value
Total number	2.1 ± 1.0	1.7 ± 1.0	0.0006
Type of medications			
ACEI or ARB	31 (51.7)	25 (41.7)	0.07
Calcium-channel blocker	51 (85)	46 (76.7)	0.125
α or β blocker	14 (23.3)	12 (20)	0.625
Diuretics, thiazide type or loop	21 (35)	16 (26.7)	0.063
Aldosterone antagonist	4 (6.7)	3 (5)	1
Other	2 (3.3)	2 (3.3)	1
Drug mitigation	–	24 (40)	–
Drug enhancement	–	4 (6.7)	–
Serum creatinine (mg/dl)	0.80 ± 0.18	0.82 ± 0.19	0.064
eGFR (ml/min/1.73 m^2^)	93.4 ± 14.7	93.0 ± 14.6	0.09

Tests of RAS	Baseline (*n* = 29)	3-month (*n* = 29)	*P* value
PRA (ng/ml/h)	4.5 ± 7.9	2.0 ± 1.6	0.01
Angiotensin II (pg/ml)	87.6 ± 20.9	76.3 ± 18.3	0.106
Aldosterone (pg/ml)	152.8 ± 39.2	149.3 ± 49.1	0.347

The results are presented as mean and standard deviation (SD), number and percent

*BP* blood pressure, *ACEI* angiotensin-converting enzyme inhibition, *ARB* angiotensin receptor blocker, *eGFR* estimated glomerular filtration rate, *RAS* renin-angiotensin system, *PRA* plasma renin activity

According to preoperative CT angiography, 27/60 (33.3%) patients had accessory renal arteries or aberrant branches supplying the treated kidneys or tumors. However, additional arteries were not all isolated in 15 cases (Table 2) for various reasons.

### Laboratory analyses

From baseline to 3 months, we observed no significant changes in serum creatinine level (0.80 ± 0.18 vs 0.82 ± 0.19 mg/dl, *P* = 0.064) or eGFR (93.4 ± 14.7 vs 93.0 ± 14.6 ml/min/1.73 m^2^, *P* = 0.09) (Table 3).

For the 29 patients not taking drugs that antagonized the renin–angiotensin system, or antiadrenergic anti-hypertensive drugs, baseline PRA was 4.5 ± 7.9 ng/ml/h as compared with 2.0 ± 1.6 ng/ml/h at 3 months after surgery, with mean change −2.5 ng/ml/h (95% CI −5.2 to −0.79; *P* = 0.01). Mean angiotensin II level was reduced by −11.3 pg/ml (95% CI −69.1 to 6.8; *P* = 0.106) and mean aldosterone level by −3.5 pg/ml (95% CI −26.6 to 9.5; *P* = 0 .347) (Table 3).

### Predictive factors

On univariable logistic regression, SBP reduction at 3 months was associated with baseline SBP [odds ratio (OR) 1.249, 95% CI 1.081–1.444, *P* = 0.003] and additional artery (OR 0.184, 95% CI 0.036–0.930, *P* = 0.041) (Table 4). We included ‘Cardiovascular disease’ (OR 2.929, 95% CI 0.692–12.401, *P* = 0.145), ‘Tumor side’ (OR 0.357, 95% CI 0.094–1.351, *P* = 0.129), ‘Baseline-SBP’ (*P* < 0.05) and ‘Additional artery’ (*P* < 0.05) in the multivariable logistic regression model. On multivariable analysis, the only independent predictor of SBP reduction was high baseline SBP (OR 1.267, 95% CI 1.076–1.491, *P* = 0.005).

**Table 4:** Univariable and multivariable analysis of predictors of SBP reduction at 3-month follow-up

Variable	Univariate			Multivariate		
	OR	95% CI	*P* value	OR	95% CI	*P* value
Gender	1.100	0.286–4.228	0.890			
Age (years)	0.989	0.930–1.051	0.718			
BMI (kg/m^2^)	1.035	0.844–1.269	0.739			
DM	1.346	0.346–5.238	0.668			
CVD	2.929	0.692–12.401	0.145	3.269	0.381–28.063	0.280
Baseline-SBP (mmHg)	1.249	1.081–1.444	0.003	1.267	1.076–1.491	0.005
HR (bpm)	0.979	0.918–1.044	0.515			
Drug	1.593	0.768–3.301	0.211			
Tumor side	0.357	0.094–1.351	0.129	0.704	0.129–3.827	0.684
Tumor size (cm)	0.911	0.632–1.312	0.616			
R.E.N.A.L score	1.225	0.892–1.681	0.209			
Tumor subtype	0.595	0.150–2.354	0.459			
Additional artery	0.184	0.036–0.930	0.041	0.183	0.027–1.258	0.084

*OR* odds ratio, *95% CI* 95% confidence interval, *BMI* body mass index, *DM* diabetes mellitus, *CVD* cardiovascular disease, *HR* heart rate, *SBP* systolic blood pressure, *R.E.N.A.L* R.E.N.A.L. Nephrometry Score

## Discussion

We wondered whether RAPN for RCC after extensive renal artery isolation affects BP in patients with poorly controlled hypertension. In this study, mean SBP and DBP decreased significantly at 3 months after surgery along with reduced requirement for antihypertensive medication. Extensive renal artery isolation during RAPN may improve BP control among patients with poorly controlled hypertension.

The effect of PN for renal masses on postoperative BP is inconsistent among several observational studies. Novick et al. evaluated the effect of PN for solitary kidneys in 14 patients, finding no changes in BP during a mean follow-up of 7.7 years [9]. In another study, PN was considered to not result in initial or long-term postoperative deterioration in BP [10]. More recently, an interesting retrospective cohort study of solitary kidney patients performed by Gupta et al. suggested that BP remains stable in the short-term and long-term after PN [11]. Conversely, Inoue et al. reported a progression of BP with PN as compared with radical nephrectomy [12]. Hutchinson et al. found PN independently associated with increased number of antihypertension medications [13]. Some case reports have indicated that PN may precipitate postoperative hypertension due to complications or “Page kidney” phenomenon [14, 15]. However, these effects may be transient if there are no renal arterial or peripheral complications [16]. More importantly, the development of hypertension is multifactorial, and other factors such as renal nerves are usually ignored. Differing from the previous PN studies, we focused on the impact of removing RSNs during RAPN.

RSNs to the kidneys terminate in the blood vessels, juxtaglomerular apparatus, and renal tubules. Stimulation of RSNs increases renin release and sodium reabsorption and reduces renal blood flow [17]. RDN was found effective for improving BP by denervating RSNs. The catheter-based RDN system emerged to selectively ablate RSNs using radiofrequency ablation. Although early results of BP control in multiple animal or human studies were encouraging, this therapeutic application has long been debated [18]. In humans, the nerves pass from the sympathetic chain and ganglia to the kidneys via the adventitia of renal arteries, leading to the hilum or just outside in peri-arterial adipose tissue and connective tissue [19]. There is a considerable distance between the arterial lumen and RSNs, which mostly locate in the tunica adventitia or surrounding tissues, especially in the proximal segment [20]. Electrodes placed inside the lumen of the artery cannot deliver radiofrequency energy effectively to all renal nerve bundles along the renal artery, especially to nerves beyond 2 mm from the lumen [21, 22].

The laparoscopic approach has been proposed to overcome the limitations of the catheter approach. Gerber et al. found that the approach was capable of removing nerves that are typically not removed by radiofrequency-based catheter ablation [23]. In a beagle model, Bai et al. reported that RDN was more effective in the adventitial group with laparotomy conducted from outside the renal artery than the intimal group with intervention from inside the artery [24]. Therefore, several laparoscopy-based RDN models were designed to ablate the renal nerves from the exterior of the renal artery [25].

Laparoscopic RDN with no ablation also shows promise in the treatment of carefully selected patients with resistant hypertension. Keisuke reported two cases of unilateral radical nephroureterectomy for renal pelvic cancer, during which the peri-arterial tissues containing RSNs were stripped to expose the vessel and ligated after cauterization; the patients showed reduced BP after surgery [26]. In our study, the renal artery was skeletonized, and adventitia around the renal artery containing RSNs was stripped off, disconnected and cauterized. Also, complete separation of the kidney further helped to remove residual sympathetic nerves that do not enter the hilum through the renal artery. Moreover, because the afferent sympathetic nerves were eliminated together with efferent nerves [19], the artery clamping or parenchyma compression effect signal from the kidney to the central nervous system by sensory pathways might be interrupted during our surgical procedure, which may be another explanation for the decreased BP.

Unilateral treatment might reduce BP if complete RDN could be achieved. One case showed that even unilateral RDN can be fully successful and decrease BP to a remarkable extent in a 12-month period [27]. Gao et al. reported considerable BP decrease with laparoscopic-based perivascular unilateral RDN [28]. In an animal model, unilateral RDN improved mean arterial pressure and cardiac autonomic balance in congestive heart failure by reducing sympathetic tone [29].

Measurements of PRA, norepinephrine spillover, or both may help in estimating the activity of the efferent RSNs. In our study, patients not on drugs antagonizing the renin–angiotensin system showed reduced PRA and angiotensin and aldosterone levels, suggesting an effect on RSNs, which was also reported in RDN studies [17]. We evaluated BP changes first at 3 months, which was considered an acceptable time for patients to observe a decrease in BP and can avoid the effects of other factors such as perioperative anesthesia, pain, fluid infusion, lifestyle changes, and seasonal changes on BP [30]. Moreover, we avoid the influence of BP optimization prior to surgery, and the medication remained unchanged during the first 3 months postoperatively, which helped in evaluating the effect of surgery.

It should be noted that the present study has some limitations. This study was terminated prematurely because most enrolled patients were lost to follow-up about 9 months postoperatively as they became reluctant, making this study a preliminary report with relatively small sample size and short follow-up time. Therefore, the finding of the present study should be interpreted carefully, and further investigations are urgently needed. In addition, there could have been a bias due to heightened self-awareness of hypertensive patients in managing blood pressure. The lack of a control arm is another drawback. Besides, we did not quantify the specific degree of RDN, for example the length of renal arterial adventitia surface, nor we took into account drug adherence, the effect of nerve fiber recovery or neuronal regeneration [17]. Moreover, to evaluate the efficacy of RDN, assessing plasma levels or kidney tissue content of norepinephrine should be valuable but was difficult to implement in our study. Furthermore, although we had tried to standardize BP measurements, ambulatory BP monitoring maybe more accurate. Last but not least, despite no attempts to perform RDN with ablation concurrently with RAPN, this combination may have a more pronounced denervation effect. For selected patients, RDN and RAPN combined may confer greater benefit beyond a decrease in BP, such as an improvement in renal function especially for solitary kidney tumors, or even oncological outcome [31]. However, further study is needed to evaluate the feasibility of this treatment mode.

## Conclusions

Our short-term findings suggest that RAPN with extensive renal artery isolation may benefit the BP of patients with poorly controlled hypertension. The removal of RSNs may play a role, but the exact mechanism and long-term effects need further investigation.

## Data Availability

The data supporting our findings are presented in the article. The datasets of the current study are available from the corresponding author on reasonable request.
